# Development of an Early Warning System to Prevent Crises in the Palliative Home Care Setting of Patients and Their Informal Caregivers: Protocol for a Mixed Method Study

**DOI:** 10.2196/13933

**Published:** 2019-11-28

**Authors:** André Fringer, Eleonore Arrer, Edith Maier, Wilfried Schnepp, Tom Ulmer

**Affiliations:** 1 Research Unit Nursing Science, Institute of Nursing Department of Health Zurich University of Applied Sciences Winterthur Switzerland; 2 Department of Nursing Science, Faculty of Health, Witten/Herdecke University Witten Germany; 3 Institute of Applied Nursing Science, FHS St Gallen, University of Applied Sciences St Gallen Switzerland; 4 Institute for Information and Process Management, FHS St Gallen, University of Applied Sciences St Gallen Switzerland

**Keywords:** palliative care, eHealth, family caregivers, outpatient care

## Abstract

**Background:**

Most people wish to die at home, but most people in Switzerland die in hospitals or nursing homes. Family caregivers often offer support so patients with palliative care needs can stay at home for as long as possible. However, crises and unplanned hospital admissions often occur in this setting because of family caregiver strain and symptom severity in patients. The so-called smart devices such as wearables or smartphones offer the opportunity to continuously monitor certain parameters and recording symptom deteriorations. By providing professionals with this information in a timely manner, crises in the home could be avoided.

**Objective:**

The aim of this interdisciplinary study is to explore the symptom burden of people with palliative care needs who are cared for at home and to understand the development of crises in the home care setting. On the basis of the findings from this study, we will develop an early warning system to stabilize the home care situation and to prevent critical events from happening, thereby reducing avoidable hospitalizations.

**Methods:**

A mixed method study is being conducted consisting of 4 main consecutive phases: (1) developing the monitoring system; (2) pretesting the system and adapting it to user needs; (3) conducting the study in the palliative home care setting with approximately 40 patients; and (4) distinguishing symptom patterns from the collected data specific to crisis emergence, followed by the development of an early warning system to prevent such crises. In study phase 3, each patient will receive an upper arm sensor and a symptom diary to assess symptom burden related to patients and family caregivers. A within-case analysis will be conducted for each patient’s situation followed by a cross-case comparison to identify certain symptom patterns that may predict symptom deterioration (study phase 4).

**Results:**

The collaboration with the local mobile palliative care team for participant recruitment and data collection has been established. Recruitment is forthcoming.

**Conclusions:**

We expect the findings of this study to provide holistic insight into symptom burden and the well-being of patients with palliative care needs and of their family caregivers. This information will be used to develop an early warning system to avoid the occurrence of potential crises, thereby improving palliative care provision at home.

**International Registered Report Identifier (IRRID):**

PRR1-10.2196/13933

## Introduction

### Background

The demand for palliative care has been increasing as a result of our ageing population and the increase in the number of chronically ill people [1,2]. Most patients wish to die in their own homes [3,4], which—because of complex and instable symptom development—is often not possible. In this regard, family caregivers represent a significant resource in the care of people at the end of life. They help ensure that patients can remain at home for as long as possible, thereby also alleviating financial strains on our health care systems [5]. The social value of family care work in Switzerland alone amounts to more than Swiss Franc 3.5 billion per year according to federal statistics [6]. Yet, family caregivers are highly vulnerable and often feel exhausted and stressed, with multiple physical and psychosocial consequences. This can lead to crises for the family or to an unplanned hospital admission for the patient [7]. In fact, patients at the end of life are especially at risk for hospitalization, thus incurring high health care costs [8,9]. A study found that 80% of severely ill patients with cancer were admitted to hospital because of uncontrolled symptoms [8]. However, such admissions could be avoided by providing adequate specialist services within home care [8,10].

Research has also found that frequent visits by health care professionals in the home are correlated with higher quality of life for family caregivers [11]. However, such teams are not always readily available and in-person visits can be costly and time consuming [12].

This is where digital health or electronic health comes in. Digital health can be defined as any “mobile health, health information technology, wearable device, telehealth, telemedicine, and personalized medicine” according to the Food and Drug Administration. In recent years, digital health has successfully been implemented within palliative care in numerous projects [13-17]. It can improve patient–health care professional interaction and optimize human resources, especially in regions where access to palliative care services is limited. In addition, and more importantly, such tools can help deliver care interventions and improve the well-being and quality of life for both patients and their families [18]. Digital technologies, such as the use of sensors for remote monitoring, can offer new opportunities for supporting family caregivers through early recognition of critical situations and preventing them from escalating. This may be achieved, for instance, by linking them up to specialized health care providers and thus bridging the geographical gap [19].

### Objectives

On the basis of these considerations, our study pursues the primary objective of exploring and understanding the home care setting of patients with palliative needs to stabilize critical situations and deflect crises. We aim to achieve this by continuously monitoring the relevant indicators and vital parameters of patients with palliative care needs in home care and thus gain an in-depth insight into individual symptom burden. At the same time, we will collect data on family caregivers’ well-being as research has shown that they can also trigger or amplify crises in the household setting and can be greatly influenced by their loved one’s illness [20,21]. This information will be used to develop an early warning system for the home care environment.

To our knowledge, no such tool has been developed that focuses on providing holistic insight into the palliative home care environment of patients and that aims at preventing the onset of crises and subsequently burdensome and costly unplanned hospital admissions.

## Methods

### Overview

The researchers have chosen to conduct a mixed method case study to investigate the different dimensions of the research topic [22].

The explorative approach is of an interdisciplinary nature and involves digital health experts, designers, and developers as well as nursing scientists and palliative care experts. Thus, it complies with international and national recommendations regarding palliative care research [23,24]. In addition, the project team is accompanied and assisted by practitioners and representatives from relevant care organizations.

The study has 4 main consecutive phases: (1) developing the monitoring system that consists of an upper arm sensor and a symptom diary, (2) pretesting the system and adapting it to user needs, (3) conducting the study in the palliative home care setting together with mobile palliative care teams (MPCTs), and (4) detecting symptom patterns from the collected data characterizing emerging crises. Finally, an early warning system to prevent such crises will be developed.

### Phase 1: Developing the Monitoring System

Before developing the monitoring system, we followed an iterative user-centered design process to ensure user acceptance. In the first phase, we conducted interviews with palliative care experts (physicians and nurses) to investigate the need and the feasibility of utilizing smart devices and symptom monitoring in the palliative home care setting. The focus of the study was primarily on the comprehensive expertise and experience of the participants. In addition, we conducted a scoping review on other monitoring systems that had been used in comparable environments.

On the basis of this preliminary work, we chose to opt for the noninvasive and nonobtrusive certified upper arm sensor from the firm Biovotion to objectively and continuously measure certain parameters automatically. These parameters include heart rate and heart rate variability, blood oxygenation, skin temperature, skin blood perfusion, respiratory rate, galvanic skin response, and micromovements. On the basis of the literature, we especially expect heart rate and respiratory rate to provide information on possible crisis situations at the end of life [25,26]. In this study, we also hope to gain more insight regarding the other parameters and their possible association with crises. The sensor itself does not indicate any information to the patient directly on a user interface but continuously transmits the data to a server after having anonymized them. The server is run by the FHS St Gallen and is hosted by a provider in Switzerland.

In addition, we designed a symptom diary for patients and their family caregivers as well as health care professionals to report the patient’s subjective symptoms 3 times per day on an intensity scale from 0 (no symptoms) to 10 (worst possible symptoms) based on the modified Edmonton Symptom Assessment System [27]. The date and time of self-symptom evaluation must be documented precisely for us to draw adequate conclusions by comparing the subjective diary data with the objective sensory data. In addition, the date will also provide us with information on the time of the year. This will be of interest for analysis purposes regarding the correlation of symptom burden or crises with, for instance, the winter months. The diary also contains the modified Caregiver Quality of Life Index—Cancer scale [28], which is filled in by the family caregiver once a week to determine the caregiver’s well-being. Even though we did not restrict our sample to patients with cancer only, this questionnaire was applied considering that most of the MPCT’s patients have cancer and that the instrument has also been used in research for family caregivers of palliative patients in general [29]. Both measuring instruments have been amended based on the feedback obtained from the expert interviews. For example, questions related to social interaction, visits by health care professionals, and overall daily burden have been added. In addition, patients and their family caregivers will be able to document their general experiences daily, including perceived crises as free text.

### Phase 2: Pretest

Before the actual study commencement, we obtained ethical approval for the study from the responsible ethics committee of Eastern Switzerland. Once obtained, we tested the monitoring system within a so-called *living laboratory* supervised by the Interdisciplinary Centre of Competence for Ageing. This pretest was conducted in an older population without severe medical conditions with the objective to make sure both the device and diary are safe, practical, and easy to use without greatly impacting the daily living experience before using them in a vulnerable population. A living laboratory can be considered an experimental natural environment where persons in their private homes participate in testing digital devices and systems. Sensory devices were given to 7 participants who also received the symptom diary. Participants with an average age of 74 years were instructed in the handling of both the sensor and the diary and were encouraged to wear the sensor continuously on 7 consecutive days. After completion of the pretest, participants’ feedback was incorporated into the development of the monitoring system and some minor adaptations were made. Overall, the participants found the sensor easy to use and said that they would be willing to wear it for a longer period than the pretest of 1 week if it was important for their health care provision. This willingness also suggests that the sensor was not bothersome to wear and did not negatively impact everyday activities. All participants wore the sensor continuously and only took it off to charge it or when showering. They also found the information on how to use the symptom diary easy to understand.

### Phase 3: Conducting the Study

#### Setting and Recruitment

Recruitment for the study is taking place in St Gallen, Switzerland. The city’s MPCT unit has a gatekeeper function and is responsible for recruiting the participants with support from the research team. We chose this approach to not disrupt this vulnerable environment of severely ill patients being cared for at home. The MPCT in St Gallen cares for at least 80 patients annually with specialized palliative care needs. Most of their patients are diagnosed with advanced cancer and are being cared for by a family caregiver in their own home.

We are planning to recruit 40 patients within a period of 12 months. Patients are eligible to participate if they are being cared for by the MPCT in St Gallen, are at least 18 years of age, speak German, and are capable of filling in the diary and using the sensor accordingly—if necessary, by receiving support from their family caregiver. The primary family caregiver is identified routinely by the MPCT as the person with the most frequent informal contact with the patient (daily visits or cohabitation requirement) and who supports the patient at home. If a patient has a pacemaker, he or she is excluded from receiving the upper arm sensor but can still fill in the symptom diary that was requested by the MPCT to facilitate patient documentation and to also provide an in-depth insight into patient and caregiver burden. We chose this specific exclusion criterion as a precautionary measure only even though we had no indication that the sensor might interfere with such a device. The diary is also given to patients who are reluctant to use the sensor at recruitment based on the consideration that they might decide to use it at a later stage after having been introduced to the diary.

#### Procedures and Data Collection

The members of the MPCT select eligible patients and decide on whether they will receive the sensor, the diary, or both (target group). This decision is based on the Karnofsky Performance Status Scale, where we distinguish between these 3 groups based on their achieved percentage scores [30]. We identify patients with a score of 50% to 60% or larger to receive the diary (group 1), 20% to 40% to receive the sensor and the diary (group 2), and 10% to receive only the sensor (group 3). The choice of categorization was based on discussions with the MPCT regarding the high vulnerability of the patient group at the end of life. It was considered to diminish the burden on those most impaired (score of 10%) together with their family caregivers by not providing the diary. Staff members have been trained by the research team on how to use the symptom diary and on how to install the sensor in the patient’s home.

Staff from the MPCT have been approaching the potential participants. Once they indicate their wish to participate, a staff member provides more information according to the allocated group and obtains informed consent. At the beginning of the study, participants also fill in a survey with questions primarily regarding the demographic data of both the patient and family caregiver (eg, age, gender, diagnosis, social contacts, and relationship with the family caregiver) to conduct subgroup analyses. The selection of these data was based on considerations to what extent they might also contribute to crises, for example, older age of the family caregiver or few social contacts. If the participant is to receive a sensor, the staff will install the device that stores the data in the patient’s home. The symptom diary will contain no information that can be tracked back to the patient, but the staff will document the diary number that also corresponds to the sensor in use. This number is essential so that we can compare the sensor data with the information documented in the diary.

The data collection period for each participant is 2 weeks, although for the entire study, data will be collected over 12 months. A time frame of 2 weeks was chosen based on considerations together with the MPCT regarding the high vulnerability of the patient group, the end-of-life situation, and the resource constraints. If a participant decides to discontinue the study before then, the data collected up to that time point will still be used for analysis, in line with the information given to the participants at recruitment. Patients (groups 1 and 2) are asked to fill in the diary 3 times daily and whenever a specific event takes place during the day that requires or indicates documentation, for instance, a crisis such as severe pain followed by specific measures. Participants of groups 2 and 3 are also asked to wear the sensor continuously during the 2 weeks and to charge it once a day (for approximately 2 hours).

After the 2 weeks of data collection, a staff member will ask the participants to fill in a survey to assess their experiences in connection with the monitoring system. Questions will, for instance, involve experiences of using the symptom diary and sensor, comfort and satisfaction, and expectations and wishes for future digital solutions. The staff member will then return the diary and the sensor to the research team, together with all other anonymous information collected.

### Phase 4: Data Analysis and Development of an Early Warning System

The data analysis will incorporate aspects of a multiple cross-case study. A within-case analysis will be conducted for each participant followed by a cross-case comparison. Primarily, this will involve comparing the data from the sensor with the symptom diary from target group 2. To do so, we will apply quantitative and qualitative methods. The content analysis method by Schreier [31] will be applied to gain insight into the caregiver’s crisis experience by analyzing the qualitative data from the diary. Two researchers will conduct the analysis using the software MAXQDA. The results will be discussed by the research team to avoid them from being influenced by the perspectives and assumptions of a single researcher. Qualitative data will later be transformed into quantitative data to compare sensor data with crisis experiences [32]. A statistician will conduct the quantitative data analysis (based on information from both the sensor and diary and the demographic data) with data comparisons, using SPSS Statistics 25 (IBM) software. Missing data, for instance, if someone forgot to fill in the diary at one point or did not wear the sensor, will be addressed by applying missing-data imputations when feasible.

We expect to be able to draw conclusions that will allow the identification of personalized threshold values that can recognize a critical event before it sets in. These threshold values will relate to sensor information, such as heart rate and respiratory rate, which we anticipate to be essential for the design of the early warning system based on previous research [25,26]. The digital warning system will be integrated in the MPCT unit and linked to a patient’s home. It will be calibrated individually for each patient including the conditions as to when the MPCT is to be alerted and the actions this will trigger.

#### Privacy and Security

To ensure participants’ privacy and to protect this vulnerable study population, the research team will rely on the MPCT to be in direct contact with the patients and families. All digitally collected information will be stored in the cloud hosted by a Swiss provider and will be anonymized in a way that it cannot be tracked back to the user. Only the research team will later have access to this information. The written information collected by the MPCT will be made anonymous before data analysis conducted by the research team.

## Results

Up to this stage, ethical clearance for the study has been obtained, the monitoring system, including the sensor and diary, has been set up, and it has been pretested in a living laboratory. The collaboration with the local MPCT for participant recruitment and data collection has been established. Recruitment is forthcoming.

## Discussion

We anticipate that our results will greatly contribute to our knowledge about the emergence of crises in the palliative care environment at home. It will be especially interesting to find out how the sensor data are correlated with the data from the symptom diary and to what extent family caregivers are stressed by events that primarily involve the patient. It is precisely this tipping point that we aim to determine, so we can intervene proactively before a crisis sets in. This can only be achieved by means of an interdisciplinary approach and in close collaboration with practitioners and patients and their family caregivers.

### Conclusions

We expect the findings of this study to provide holistic insight into the symptom burden and well-being of patients with palliative care needs and of their family caregivers. This information will be used to develop an early warning system to avoid the occurrence of crises within home care. Future provision of palliative care by professionals in a patient’s home could thus be improved. Given the future challenges connected to demographic change, staff shortages, or economic pressures, the proposed early warning system has the potential to alleviate these pressures to some extent by complementing and enhancing patient–health care professional interaction and stabilizing the home care environment.

## Abbreviations

MPCT: mobile palliative care team
